# Supplementary material for: Inducible miR-150 Inhibits Porcine Reproductive and Respiratory Syndrome Virus Replication by Targeting Viral Genome and Suppressor of Cytokine Signaling 1
Source: Viruses. 2022 Jul 7;14(7):1485. doi: 10.3390/v14071485 (PMC9318191; doi:10.3390/v14071485)
Supplement: Supplementary file 1 [file viruses-14-01485-s001.zip › Table S1.pdf]

Table S1 Sequences of miRNA and siRNA

name	sequence
siSOCS1-1	CCUGCACGGAGCAUUAACUTT
siSOCS1-2	GCCGACAAUGCAAUCUCCATT
sic-Jun	GCAAAGAUGGAAACGACCUTT
ssc-miR-150	UCUCCCAACCCUUGUACCAGUG
ssc-miR-150 inhibitor	CACUGGUACAAGGGUUGGGAGA
