# Supplementary material for: Inducible miR-150 Inhibits Porcine Reproductive and Respiratory Syndrome Virus Replication by Targeting Viral Genome and Suppressor of Cytokine Signaling 1
Source: Viruses. 2022 Jul 7;14(7):1485. doi: 10.3390/v14071485 (PMC9318191; doi:10.3390/v14071485)
Supplement: Supplementary file 1 [file viruses-14-01485-s001.zip › Table S2.pdf]

Table S2 Primers used in qRT-PCR analysis

Primers	Sequences (5'→3')
ORF7-F	AATAACAACGGCAAGCAGCA
ORF7-R	GCACAGTATGATGCGTCGGC
MX1-F	CACAGAACTGCCAAGTCCAA
MX1-F	GCAGTACACGATCTGCTCCA
ISG56-F	TCAGAGGTGAGA AGGCTGGT
ISG56-R	GCTTCCTGCAAGTGTCCTTC
SOCS1-F	CGCCCTCAGTGTGAAGATGG
SOCS1-R	GCTCGAAGAGGCAGTCGAAG
GAPDH-F	CCTTCCGTGTCCCTACTGCCAAC
GAPDH-R	GACGCCTGCTTCACCACCTTCT
PEDV-N-F	AACAGCTTCCCAGCGTAGTTGA
PEDV-N-R	GAAGTGGCTCTGGATTTGTTCTTC
miR-150-F	AGTGCTGTCTCCCAACCCTT
miR-150-R	TATGGTTGTTCACGACTCCTTCAC
U6-F	CGCTTCGGCAGCACATATAC
U6-R	TTCACGAATTTGCGTGTCATC
HV UTR-F	TGGCATTCTTTGGCACCTCA
HV UTR-R	ATTACGGCCGCATGGTTCTC
