# Supplementary material for: Inducible miR-150 Inhibits Porcine Reproductive and Respiratory Syndrome Virus Replication by Targeting Viral Genome and Suppressor of Cytokine Signaling 1
Source: Viruses. 2022 Jul 7;14(7):1485. doi: 10.3390/v14071485 (PMC9318191; doi:10.3390/v14071485)
Supplement: Supplementary file 1 [file viruses-14-01485-s001.zip › Table S3.pdf]

Table S3 Primers used in PCR and plasmid construction

Primers	Sequences (5'→3')
-2029/+25 F	GGCTAGCCATCACACCCAAGGTCACCCA
-1524/+25 F	GGCTAGCCTGCCCAAGTCCCTTCATAAACA
-1014/+25 F	GGCTAGCCTGGGAGAATGTGGGGATTGA
-500/+25 F	GGCTAGCCACCGGGGCTATCAGGTGT
-43/+25 F	GGCTAGCCCGGGCCGGTCCAGAGGCA
R	CCTCGAGGACAAGGGTTGGGAGATAGG
PRRSV 3'UTR-F	GCTCTAGATGGGCTGGCATTCTTTGG
PRRSV 3'UTR-R	GCTCTAGAAATTACGGCCGCATGGTTCT
PRRSV 3'UTR-mut R	CTCTATGTGTGGTGAATGGCAC
PRRSV 3'UTR-mut R	GGTTTTCTAACACTGAGGTGCC
SOCS1 3'UTR F	CTCTAGAGCTGCACGGAGCATTAACT
SOCS1 3'UTR R	CTCTAGAGTTATTACCTAAACTGGTGGT
SOCS1 3'UTR-mut F	TGTAGGGTCTCTGGCTTTATTTTTC
SOCS1 3'UTR-mut R	AACCCCCCTGGTTTGTGCAAAG
ChIP c-Jun1-F	ACCTGAGACTAGGGAGCCTGA
ChIP c-Jun1-R	GTGGGGCTGCCGTGTTTAC
ChIP c-Jun2-F	GGTCTCTGTCCCTCTCGGA
ChIP c-Jun2-F	CAAAGAGGGCAGGAAGGGG
